# An Insight Into the Effect of Odontogenic Keratocysts on Surrounding Structures: Cone-Beam Computed Tomography-Based Analysis of Cases

**DOI:** 10.7759/cureus.40488

**Published:** 2023-06-15

**Authors:** Varsha AC, Ajay Parihar, Ashish Saxena

**Affiliations:** 1 Department of Oral Medicine and Radiology, Government College of Dentistry, Indore, IND; 2 Department of Pediatric and Preventive Dentistry, Government College of Dentistry, Indore, IND

**Keywords:** unilocular, odontogenic keratocyst, multilocular, maxilla, mandible, cone-beam computed tomography

## Abstract

Introduction

An odontogenic keratocyst (OKC) is a benign intraosseous lesion with potential to demonstrate aggressive and invasive behavior. The aim of this retrospective study was to analyze the imaging features of the OKC using cone-beam computed tomography (CBCT) and to evaluate the association between the internal structure of the lesion and the effect of the lesion on surrounding structures.

Methods

Overall, 32 CBCT scans of histopathologically diagnosed cases of OKC were analyzed retrospectively. The following variables were analyzed: anatomic location of the lesions (mandible body (right/left), ramus (right/left), mandible body+ramus (right/left), maxilla (right/left), and both jaws), the internal structure of the lesion (unilocular/multilocular), and the effect of the lesion on the surrounding anatomical structures (involvement of the inferior alveolar nerve canal (IANC), displacement of the IANC, cortical expansion, displacement of the tooth, resorption of the root, associated impacted tooth, associated missing tooth). We also looked for the association between the internal structure and the effect of the lesion on anatomic structures.

Results

Out of 32 cases, 29 (90.6%) cases involved the mandible alone. Statistically significant association (p-value 0.005) was present between the internal structure and mean age of presentation as well as between the internal structure and impacted tooth (p-value 0.027). The association between the internal structure and other variables was statistically not significant.

Conclusions

The radiographic features of OKCs can be variable, and these lesions have a considerable effect on the tooth, IANC, and cortical bone. Significant association was found between the internal structure, age, and impacted tooth. Since OKCs have a high recurrence rate, CBCT is advised for evaluating the extent and location of any cortical perforations.

## Introduction

An odontogenic keratocyst (OKC) is a benign intraosseous lesion that is derived from the remnants (rest) of the dental lamina. The term ‘odontogenic keratocyst’ was first used by Philipsen in 1956, while the essential features of this type of cyst were described by Pindborg and Hansen in 1963. Histologically, OKCs originate from the dental lamina and the cystic space that contains desquamated keratin and are lined with a uniform parakeratinised squamous epithelium of 5 to 10 cell layers. They are unique odontogenic lesions that have the potential to behave aggressively, can recur, and can be associated with the nevoid basal cell carcinoma syndrome [1].

OKCs commonly originate in the tooth-bearing areas of the jaw and most commonly arise in the posterior body of the mandible and mandibular ramus. Occasionally, OKCs may develop in association with the crown of an unerupted or impacted tooth, making it difficult to distinguish them from dentigerous cysts [2].

Various imaging modalities are available for radiological evaluation including panoramic radiographs and three-dimensional modalities like computed tomography and cone-beam computed tomography (CBCT). CBCT provides high spatial resolution, accurate three‑dimensional representation of jaw lesions, borders of large lesions, bone expansion, and cortical perforation with a relatively low dose of radiation [3].

In this study, we analyzed the imaging features of OKCs using CBCT. The aim of this study is to analyze the effect of OKCs on surrounding anatomic structures and to evaluate the relation between the internal structure of the lesions and the effect on surrounding structures.

## Materials and methods

CBCT volumes of 32 cases of OKCs were analyzed retrospectively. Data were collected from the archives of various imaging centers in Madhya Pradesh reported during 2019-2022. The study included data of histopathologically diagnosed cases of OKC and CBCT volumes of adequate field of view and image quality. For all the cases, demographic data including age and gender were recorded. The anatomic location of the lesions included the mandible body (right/left), ramus (right/left), mandible body+ramus (right/left), maxilla (right/left), and both jaws. Based on the presence or absence of septations, the internal structure of the lesions was divided into unilocular and multilocular. The effect of the lesion on the surrounding anatomical structures included involvement of the inferior alveolar nerve canal (IANC), displacement of the IANC, cortical expansion, displacement of the tooth, resorption of the root, associated impacted tooth, and associated missing tooth. We also looked for the association between the internal structure and the effect of the lesion on anatomic structures and demographic data.

The study excluded cases without a histopathological report, CBCT volumes with poor quality/blurred images, and data in which metal artifacts were obscuring the view.

The data were analyzed using CS 3D Imaging software (CS 3D imaging v3.10.4). The coronal and sagittal planes were adjusted so that the maximum width and clear view of the pathology were obtained. Axial view analysis was done to confirm the presence or absence of expansion. The brightness and contrast were also adjusted using the software’s multiplanar reformatted view.

Statistical analysis

The data were collected and entered into Microsoft Excel 2016. Data were analyzed using IBM SPSS Statistics for Windows, Version 15 (Released 2006; IBM Corp., Armonk, New York, United States). The collected data were summarized using descriptive statistics (e.g., mean, standard deviation, frequency, percentage). The independent t-test was used to analyze the differences between continuous independent variables, and categorical variables were analyzed using Chi-square and Fisher’s exact test, as appropriate. The confidence interval was set to 95% and p-values less than 0.05 were considered as significant.

## Results

The study sample included 19 (59.37%) males and 13 (40.62%) females. The mean age of the patients was 35.75 years (range: 11-69 years). Out of 32 cases, 29 (90.6%) of the cases involved the mandible alone, one case involved the maxilla alone, and two cases involved both the maxilla and mandible. In the mandible, 15 (46.8%) cases involved both the ramus and mandibular body, 13 (40.62%) cases involved the mandibular body alone and three (9.35%) cases involved the ramus alone. Two lesions in the mandible were found to be crossing the midline. The demographic data and anatomic location of the lesion are summarized in Table 1.

**Table 1:** Demographic data and anatomic location of the lesion

No	Age	Sex	Anatomic location				
			Mandible body (right/left)	Ramus (right/left)	Body+ramus (right/left)	Maxilla (right/left)	Both jaws
1	52	Male	No	No	Right	No	No
2	50	Male	Left	No	No	No	No
3	45	Male	Right	No	No	No	No
4	42	Male	No	No	No	Bilateral	No
5	18	Female	Right	No	No	No	No
6	11	Female	No	No	Bilateral	Right	Yes
7	39	Female	Left, midline	No	No	No	No
8	35	Female	No	No	Left	No	No
9	33	Male	Left	No	No	No	No
10	29	Female	No	No	Left	No	No
11	65	Male	No	No	Right	No	No
12	25	Male	No	No	Bilateral	Right	Yes
13	25	Female	No	No	Left	No	No
14	69	Male	Right	No	No	No	No
15	52	Male	No	No	Yes	No	No
16	45	Female	Right	No	No	No	No
17	17	Male	No	No	Right	No	No
18	50	Male	No	No	Right	No	No
19	12	Female	No	No	Left	No	No
20	16	Female	Right	No	No	No	No
21	42	Male	Left	No	No	No	No
22	50	Male	Left	No	No	No	No
23	16	Male	Left	No	No	No	No
24	29	Female	Left	No	No	No	No
25	30	Female	Anterior body	No	No	No	No
26	35	Male	No	Yes	No	No	No
27	23	Male	No	No	Right	No	No
28	40	Male	No	Left	No	No	No
29	27	Female	No	No	Right	No	No
30	30	Female	No	No	Left	No	No
31	32	Male	No	No	Left	No	No
32	60	Male	No	Left	No	No	No

**Figure 1:**
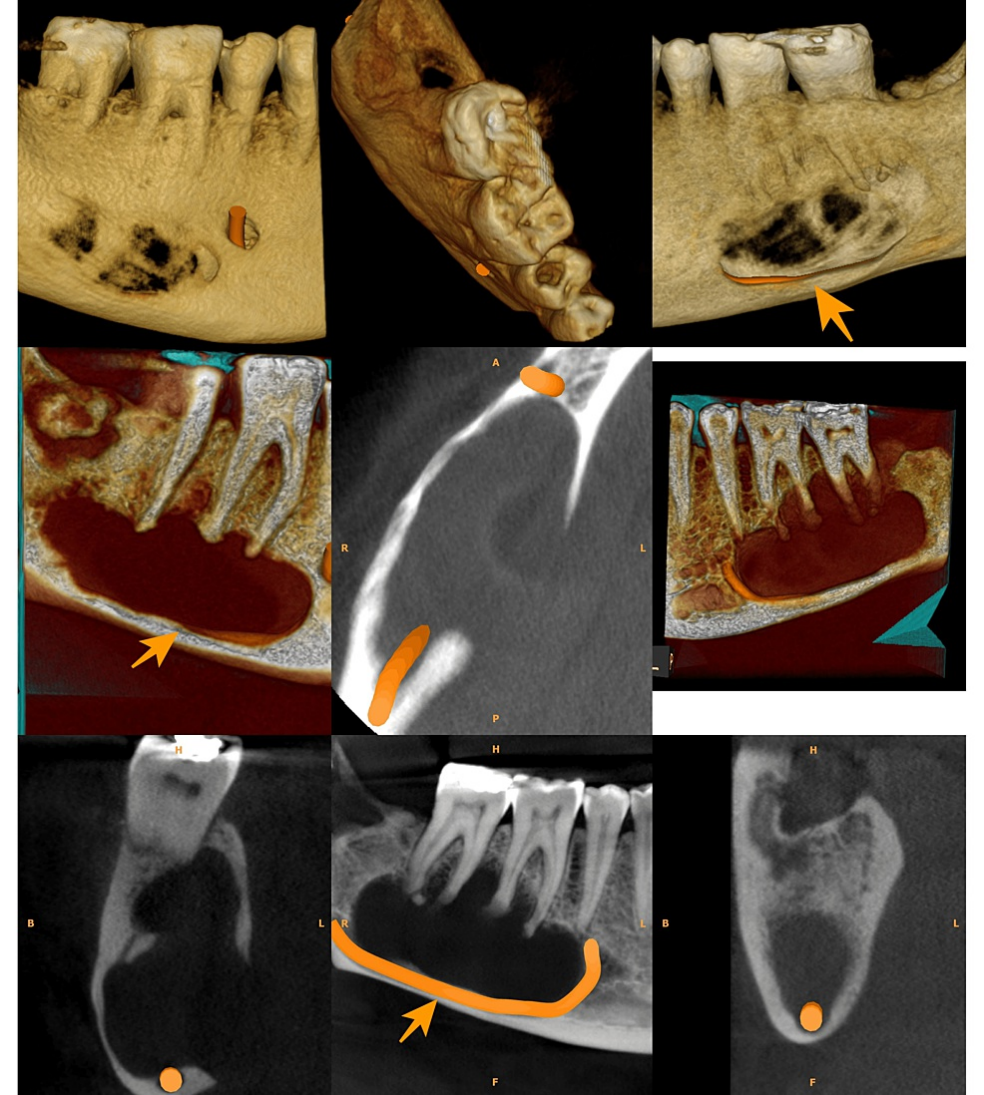
OKC involving the body of the mandible causing inferior displacement of the IANC OKC: Odontogenic keratocyst; IANC: inferior alveolar nerve canal

Of all the cases, 15 (46.9%) cases were unilocular and 17 (53.1%) were multilocular. The lesion caused displacement of the IANC in 21 (65.6%) cases (Figure 1).

**Figure 2:**
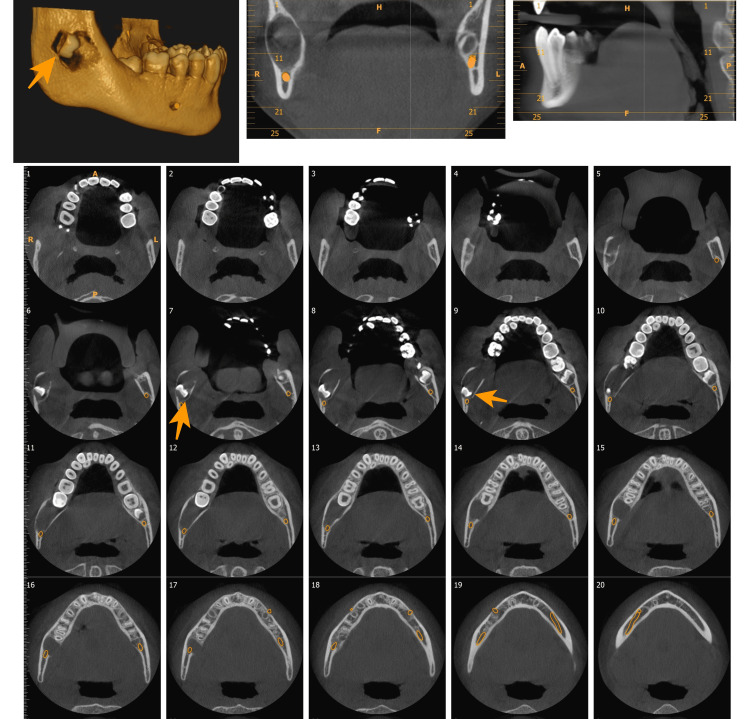
Unilocular lesion associated with impacted 48 causing posterior and superior displacement of the tooth.

Only one (13.1%) case showed involvement of the IANC. In seven (21.9%) cases, the nerve canal was not traceable. When it comes to the effect of the lesion on surrounding teeth, seven (21.9%) cases were associated with missing tooth and 10 (31.3%) cases were associated with the impacted tooth (Figure 2).

**Figure 3:**
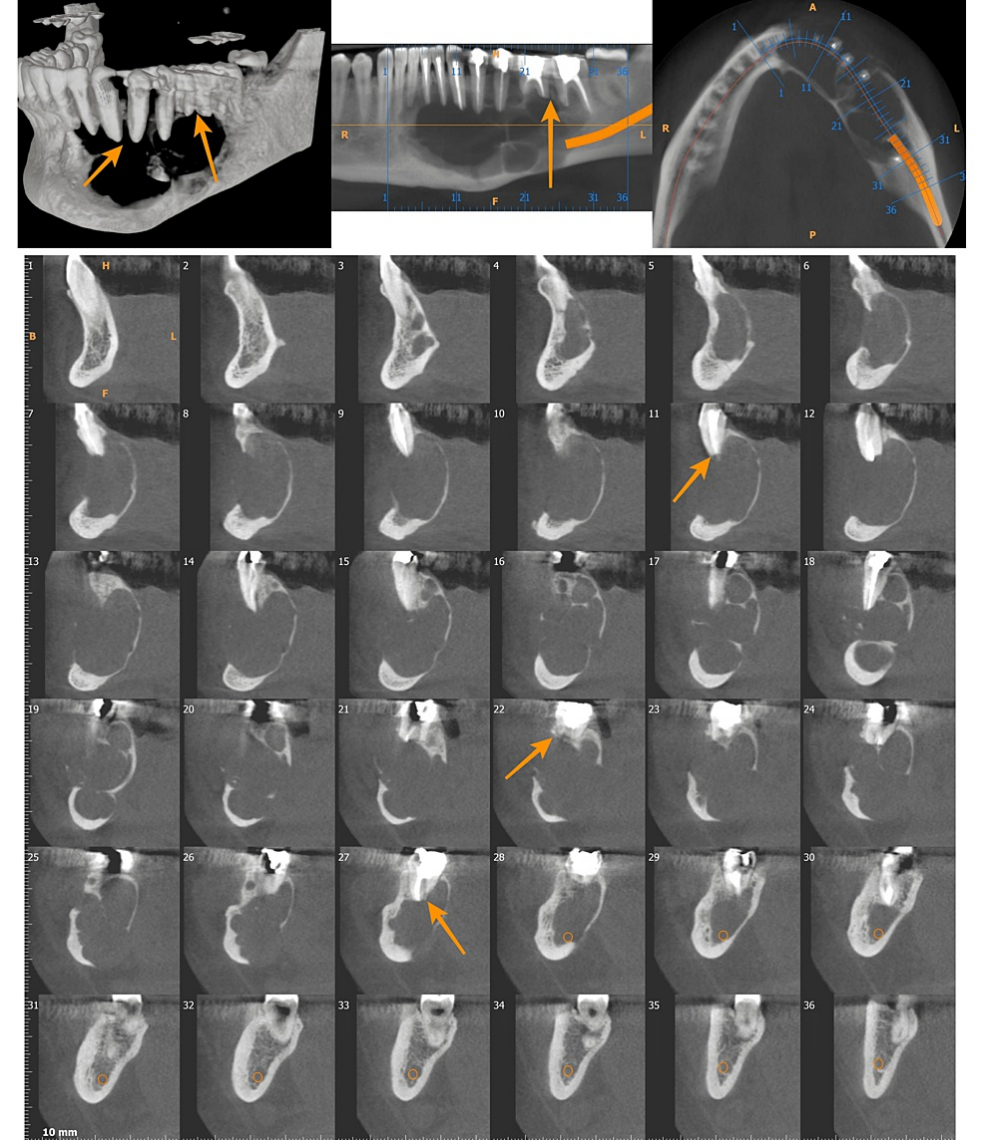
OKC with a multilocular pattern showing root resorption of 33, 34, 35, 36, and 37

Of all the cases, six (18.8%) cases caused displacement of the tooth and 10 (31.3%) cases were associated with root resorption (Figure 3).

**Figure 4:**
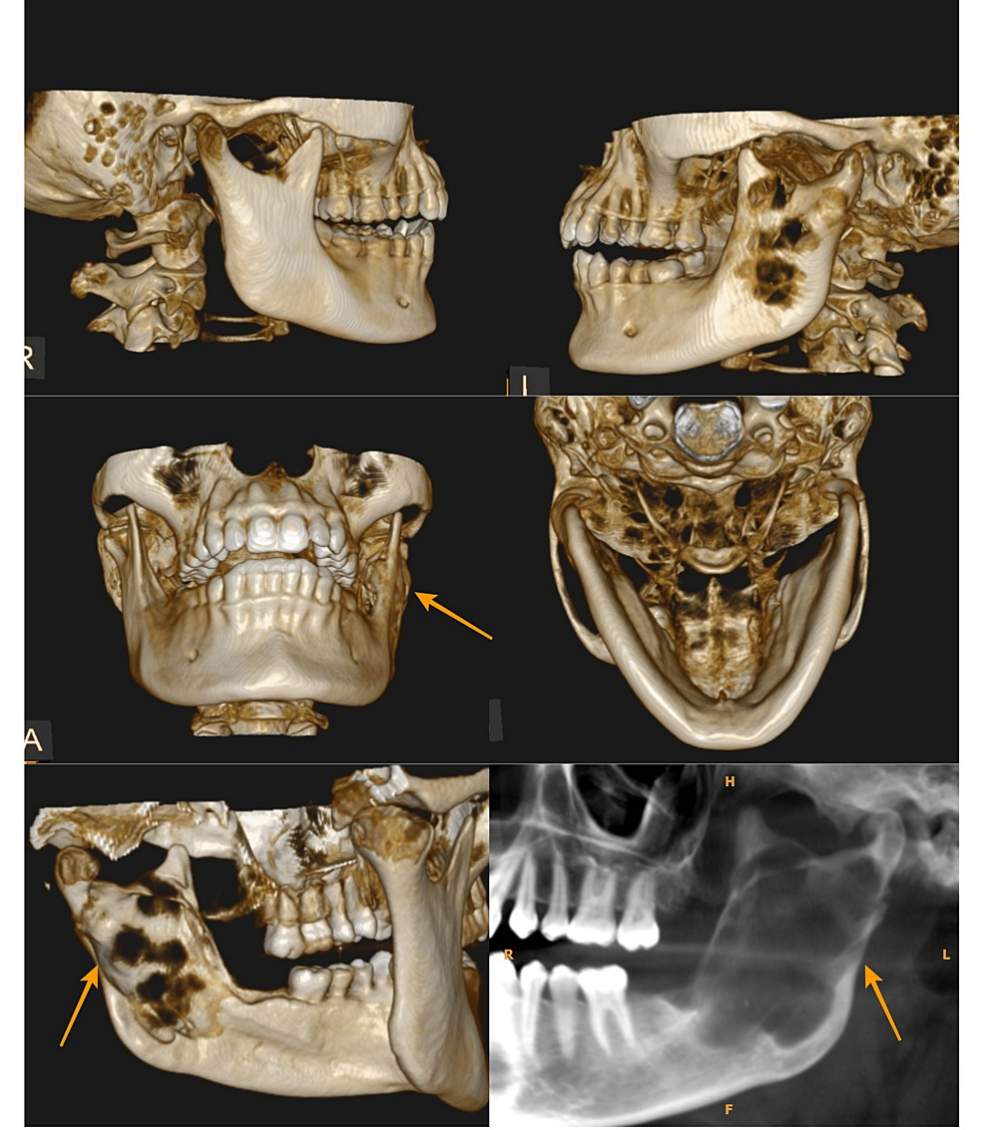
Multilocular lesion involving the right ramus of the mandible showing minimal expansion of bone irrespective of the size of the lesion

The cortical expansion was seen in all the cases analyzed in this study, but half of the cases showed minimal expansion only (Figure 4).

The effect of lesions on anatomic structures is demonstrated in Table 2.

**Table 2:** Effect of the lesion on surrounding anatomic structures

No	Involvement of nerve canal (yes/no)	Displacement of nerve canal (yes/no)	Displacement of tooth (yes/no)	Resorption of root (yes/no)	Associated missing tooth (yes/no)	Impacted tooth (yes/no)	Cortical expansion	Internal structure
1	Yes	No	No	No	No	No	Yes, minimum	Multilocular
2	Not traceable	Not traceable	No	Yes	No	No	Yes	Multilocular
3	No	Yes	No	No	No	No	Yes	Multilocular
4	No	No	No	No	No	No	Yes	Multilocular
5	No	Yes	Yes	Yes	Yes	No	Yes	Multilocular
6	No	Yes	Yes	Yes	No	Yes	Yes, minimum	Unilocular
7	No	Yes	No	Yes	No	No	Yes	Multilocular
8	No	Yes	No	No	No	No	Yes	Multilocular
9	No	Yes	No	No	No	Yes	Yes, minimum	Unilocular
10	No	Yes	No	No	No	Yes	Yes, minimum	Multilocular
11	Not traceable	Not traceable	No	No	No	No	Yes	Multilocular
12	Not traceable	Not traceable	No	No	Yes	Yes	Yes	Multilocular
13	No	Yes	No	Yes	Yes	No	Yes	Multilocular
14	Not traceable	Not traceable	No	No	No	No	Yes, minimum	Multilocular
15	No	No	No	No	No	No	Yes, minimum	Multilocular
16	No	Yes	No	Yes	No	No	Yes, minimum	Multilocular
17	Not traceable	Not traceable	Yes	Yes	No	Yes	Yes	Unilocular
18	Not traceable	Not traceable	No	No	No	No	Yes, minimum	Unilocular
19	No	Yes	Yes	No	No	Yes	Yes, minimum	Unilocular
20	No	Yes	No	Yes	No	No	Yes	Unilocular
21	No	Yes	Yes	Yes	No	No	Yes	Multilocular
22	No	Yes	Yes	Yes	Yes	No	Yes, minimum	Unilocular
23	No	Yes	No	No	No	No	Yes, minimum	Unilocular
24	No	Yes	No	No	No	Yes	Yes, minimum	Uniilocular
25	No	No	No	No	Yes	Yes	Yes	Multilocular
26	Not traceable	Not traceable	No	No	No	No	Yes, minimum	Unilocular
27	No	Yes	No	No	No	Yes	Yes	Unilocular
28	No	Yes	No	No	No	No	Yes, minimum	Unilocular
29	No	Yes	No	No	Yes	Yes	Yes	Unilocular
30	No	Yes	No	No	No	Yes	Yes	Unilocular
31	No	Yes	No	No	Yes	Yes	Yes, minimum	Unilocular
32	No	Yes	No	No	No	No	Yes, minimum	Multilocular

The present study also analyzed the association of the internal structure of OKCs with other variables (Table 3).

**Table 3:** Association between the internal structure of lesions and the effect of lesions on surrounding anatomic structures a: Independent t test; b: Fisher's exact test; c: Chi-square test; *: statistically significant at p<0.05

Internal Structure	Age (Mean ± SD)	Gender		Involvement of Nerve Canal		Displacement of the Nerve Canal				Displacement of Tooth		Resorption of Root		Associated Missing Tooth		Impacted Tooth		Cortical Expansion		
		Male	Female	Yes	No	Not Traceable	Yes	No	Not Traceable	Yes	No	Yes	No	Yes	No	Yes	No	Yes	Yes, Minimum	
Unilocular	28.07 ± 12.50	9 (60)	6 (40)	0 (0)	12 (80)	3 (20)	12 (80)	0 (0)	3 (20)	4 (26.6)	11 (73.3)	4 (26.6)	11 (73.3)	3 (42.8)	12 (48)	9 (75)	6 (30)	5 (31.2)	10 (62.5)	
Multilocular	42.53 ± 14.54	10 (58.8)	7 (41.1)	1 (5.8)	12 (70.5)	4 (23.5)	9 (52.9)	4 (23.5)	4 (23.5)	2 (11.7)	15 (88.2)	6 (35.2)	11 (64.7)	4 (57.1)	13 (52)	3 (25)	14 (70)	11 (68.7)	6 (37.5)	
p-value	0.005^a^	0.615^b^		0.600^c^		0.107^c^				0.383^b^		0.712^b^		0.576^b^		0.027*^b^		0.156^b^		

The mean age of presentation of the lesions with a unilocular structure was 28.07 ± 12.50 years, whereas the multilocular variant showed a more older presentation (42.53 ± 14.54). Slight predilection for male gender was shown by both unilocular (60%vs40%) and multilocular (58.8vs1.1) variants, and the association was statistically not significant (p-value 0.615). The involvement of the IANC was showed by a single case which showed a multilocular internal structure, and the majority of multilocular (70.5%) and unilocular (80%) lesions did not show the involvement of nerve canal. Among unilocular and multilocular lesions, 80% of the unilocular lesions and 52.9% of the multilocular lesions caused displacement of the IANC, and this association was statistically not significant (p-value 0.107). The nerve canal was not traceable in 20% of unilocular cases and 23.5% of multilocular cases.

We found statistically significant association (p-value 0.027) between the internal structure and impacted tooth, where 75% of unilocular lesions and 25% of multilocular lesions were associated with the impacted tooth. The association between the internal structure and displacement of tooth was non-significant, in which the majority of unilocular (73.3%) and multilocular (88.2%) lesions did not show association. Among unilocular and multilocular lesions, only 26.6% of unilocular lesions and 35.2% of multilocular lesions were associated with resorption of the root, and the association was statistically not significant (p-value 0.712). The association between the missing tooth and internal structure was not significant (p-value 0.576), where 42.8% of unilocular lesions and 57.1% of multilocular lesions showed association. Even though every case discussed showed cortical expansion, 62.5% of unilocular cases and 37.5% of multilocular cases showed minimal expansion and considerable expansion was shown by 31.2% of unilocular cases and 68.7% of multilocular cases and this association was statistically not significant (p-value 0.156).

## Discussion

OKCs show a considerably wide range of age distribution (from 8 to 82 years), with a peak of incidence in the third decade of life [4-6]. The results of the present study also unequivocally point to a broad range of age distribution with a mean age of 35.75 years. Male predominance was reported by various studies similar to the current study [7].

Even though OKCs can occur anywhere in the jaws, the majority occur in the mandible [8], and in the mandible, they most commonly arise in the posterior body (90% occur distal to the canine teeth) and mandibular ramus (>50%) [2] which was similar to the present study where in the mandible only one case solely involved anterior body, one case involved both anterior and posterior body and all other cases involved posterior body.

OKCs can also have ample effects on surrounding anatomic structures like teeth, IANC, and cortical bone. Mortazavi et al. analyzed various odontogenic lesions causing displacement of the IANC and found that 53.8% of OKCs caused buccal displacement and 46.2% caused lingual displacement of the IANC [9,10]. Similar to this, we found a considerable effect of OKCs on the IANC with 65.6% of cases causing displacement irrespective of the direction of displacement of the canal.

An important characteristic of the OKC is minimal buccolingual expansion assuming a fusiform shape rather than the balloon expansion which is mostly seen in the ameloblastoma [3]. Even though all cases discussed above showed bony expansion, half of the lesions showed minimal expansion, supporting the literature.

The typical radiographic features of OKCs include unilocular, multilocular, or multiple well-circumscribed radiolucent lesions surrounded by a thin radiopaque border with a smooth or loculated periphery [11]. Some previous studies have reported the predominance of unilocular over multilocular [12,13]. In contrast to this, our study showed slight predominance of the multilocular pattern (53.12%) and all multilocular lesions showed a soap bubble appearance.

Our study also evaluated the association of the internal structure of the lesion with its effect on surrounding anatomic structures and patient’s demographic data. To the best of our knowledge, this is the first study which analyzed the association between all these variables.

Macdonald-Jankowski et al. analyzed 33 cases of OKCs among the Hong Kong population and reported that in older patients multilocular lesions could be found more than unilocular ones [14]. Similar to this report, the younger mean age of presentation was showed by lesions with a unilocular pattern (28.07 ± 12.50 years), whereas the multilocular variant showed a more older presentation (42.53 ± 14.54 years) and this association was found to be statistically significant (p <0.05).

In the literature, various studies are available analyzing the association between odontogenic lesions and impacted tooth [15] and 25%-40% of the lesions of OKCs co-occur with an impacted tooth [16]. Karabas et al. conducted a retrospective study analyzing radiolucent lesions associated with impacted tooth using CBCT and found that the most common lesions associated were dentigerous cysts (60%) and OKCs (26.3%), but the authors did not differentiate the association between the internal structure of the lesion and impacted tooth [17]. In our study, 31.3% of cases were associated with impacted tooth similar to the literature and we found statistically significant association between the internal structure of the lesion (p <0.05) and impacted tooth.

Previous literature studies have reported the association between OKCs and displacement of adjacent tooth, stating that 17-100% of OKCs showed tooth displacement [13,16-19]. The present study results were also in the same range (18.8%), but they did not show statistically significant association with the internal structure of the lesion (p >0.05). The lesions of OKCs can also cause tooth root resorption rarely and this rate was reported to be 8%-41%, which was similar to the current study where 31.3% of the cases were causing root resorption [13,20].

**Figure 5:**
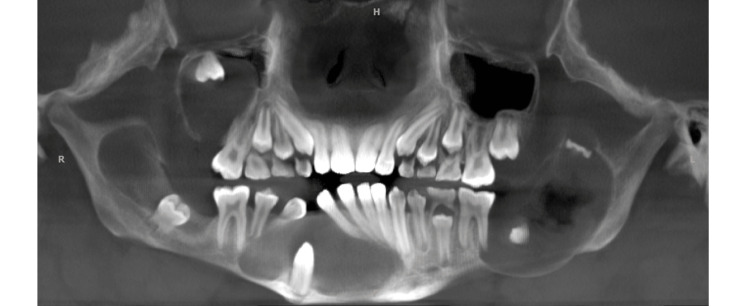
A case of Gorlin-Goltz syndrome showing multiple lesions of OKCs involving both jaws OKCs: Odontogenic keratocysts

OKCs have two broad clinical manifestations, as solitary (sporadic) or syndromic [21]. According to a systematic review, the most common variant is solitary accounting for nearly 94% of all OKCs [22]. Multiple OKCs can be seen associated with Gorlin-Goltz syndrome, which is also known as nevoid basal cell carcinoma syndrome. This is a rare multisystemic disease with a high degree of variable expressiveness and penetrance [23,24]. This condition arises from the mutation of tumor suppressor gene called patched (PTCH) and can be transmitted in an autosomal dominant way or it can also arise from a spontaneous mutation [20,21]. Out of two cases presented with multiple lesions of OKCs mentioned in this study, one patient was diagnosed to have Gorlin-Goltz syndrome (Figure 5).

OKCs are considered an aggressive benign neoplasm of the jaws with a high recurrence rate and the recurrence rate varies from 12% to 58% [13,25-28]. Recurrent lesions usually develop within the first five years but may occur as much as 10 years later [2], and multilocular lesions were reported to show recurrence more frequently than unilocular lesions [29]. Since cortical perforation, a large size, and daughter cysts are few clinicopathological features associated with a high recurrence rate and three-dimensional imaging modalities like CBCT give a clear idea about size and bony perforations, the use of CBCT is indicated for evaluating the recurrence of OKCs [30].

Even though panoramic radiographs are useful for screening and gross evaluation of maxillofacial structures, there are various factors which limits their usage in evaluating the exact imaging features including geometric distortion, lack of fine details, and numerous artifacts [31]. Compared to other imaging modalities, CBCT is superior as it gives accurate and faster three‑dimensional representation of a lesion at a lower dose, cost, and minimal distortion. So CBCT is highly recommended for evaluating the extent and imaging features of OKCs as these features play a vital role in their management and in predicting recurrence. 

Limitations of the study

The major limitation of this study is the small sample size. Other limitations are that the anatomical location of the lesion involving the maxilla was not classified as in the mandible since only very few cases involved the maxilla and the size of the lesions was not measured.

## Conclusions

The radiographic features of OKCs can be variable, and these lesions have a considerable effect on tooth, inferior alveolar nerve canal, and cortical bone. Significant association was found between the internal structure, age, and impacted tooth. Due to the high recurrence rate of OKCs and increased association of multilocular pattern, size, and cortical perforation with the recurrence rate, CBCT is advised for evaluating the exact internal structure and extent of the lesion.
